# Effects of in Season Multi-Directional Plyometric Training on Vertical Jump Performance, Change of Direction Speed and Dynamic Postural Control in U-21 Soccer Players

**DOI:** 10.3389/fphys.2020.00374

**Published:** 2020-04-30

**Authors:** Mohamed Chedly Jlid, Jérémy Coquart, Nicola Maffulli, Thierry Paillard, Gian Nicola Bisciotti, Karim Chamari

**Affiliations:** ^1^Research Unit (UR17JS01) Sport Performance, Health and Society, Higher Institute of Sport and Physical Education of Ksar Said, University of Manouba, Tunis, Tunisia; ^2^CETAPS, EA3832, UFR STAPS, Université de Rouen Normandie, Rouen, France; ^3^Centre for Sports and Exercise Medicine, Mile End Hospital, Barts and The London School of Medicine and Dentistry, London, United Kingdom; ^4^Laboratoire Mouvement, Equilibre, Performance et Santé (EA 4445), Département STAPS, Université de Pau et des Pays de l’Adour, Tarbes, France; ^5^Athlete Health and Performance Research Centre, ASPETAR, Qatar Orthopaedic and Sports Medicine Hospital, Doha, Qatar

**Keywords:** plyometric training, agility, balance, soccer, strength

## Abstract

The aim of the study was to assess the effects of multi-directional plyometric training (MPT) on vertical jump height, change of direction speed (CODS), and dynamic postural control (DPC) of soccer players under 21 year (U-21). Twenty-seven male soccer players were randomly allocated to either an experimental group (EG; *n* = 14; age: 19.0 ± 0.9 years) or a control group (CG; *n* = 13; age: 19.0 ± 0.7 years). The EG introduced 6 weeks MPT, 2 days per week into their in-season training, while CG continued training without change. Measurements of vertical jump height, CODS and DPC were completed at the beginning and end of the 6 week MPT. ANOVA demonstrated a significant group × time interaction for SJ (*F* = 6.03, *p* = 0.021), CMJ (*F* = 9.10, *p* = 0.006), and *T*-Test (*F* = 10.46, *p* = 0.002). The Bonferroni *Post Hoc* test demonstrated significant increase for the three tests in both group (EG and CG). For SJ (EG: *p* < 0.001; CG: *p* < 0.001), CMJ (EG: *p* < 0.001; CG: *p* = 0.005) and *T*-Test (EG: *p* < 0.001; CG: *p* = 0.02). For DPC on the dominant leg, there was a significant group × time interaction for four axes [anterior (*F* = 5.48, *p* = 0.028), antero-lateral (*F* = 4.82, *p* = 0.038), postero-lateral (*F* = 4.82, *p* = 0.038), and medial (*F* = 6.77, *p* = 0.015)]. The Bonferroni *Post Hoc* test demonstrated significant increase in EG (*p* < 0.001), but no significant change in CG in four axes (anterior, antero-lateral, postero-lateral and medial). Furthermore DPC on the non-dominant leg, there was a significant group × time interaction for three axes [lateral (*F* = 8.09, *p* = 0.009), postero-lateral (*F* = 11.92, *p* = 0.002), and medial (*F* = 5.84, *p* = 0.023)]. The Bonferroni *Post Hoc* test demonstrated significant increase in EG (*p* < 0.001), but no significant change in CG in three axes (lateral, postero-lateral, and medial). In conclusion, incorporating MPT into the in-season regimen of under 21 soccer players improved performance of various indices related to soccer activity (i.e., CMJ, CODS, and DPC). MPT has the potential to be appealing to coaches, as it requires little time while yielding valuable results in the physical preparation of soccer players.

## Introduction

In soccer, the ability to perform such rapid actions as sprinting, jumping, kicking, and changing direction are essential to optimizing the chances of winning the match (Reilly et al., 2000; Little and Williams, 2005). Many soccer-specific movements as displacements at high speed are characterized by a succession of rapid eccentric and high-velocity concentric muscular contractions, involving the stretch-shortening cycle (SSC) (Markovic and Mikulic, 2010; Bedoya et al., 2015). Sarmento et al. (2014) present studies regarding time-motion analysis on soccer. The studies were grouped by movement categories according to their intensity, ranging from five to seven categories from “standing” to “sprinting,” trying to characterize the physical requirements in football. In general, these studies have shown that elite players normally covered distances between 9 and 14 km, and performed approximately 1330 activities during a match, including 220 displacements at high speed (Barros et al., 2007; Di Salvo et al., 2007; Rampinini et al., 2007). Plyometric training (PT) is used to improve exercise performance that involves SSC of muscle–tendon units (Markovic and Mikulic, 2010). Therefore, PT may be considered as an appropriate training stimulus to developing explosive strength (Ramirez-Campillo et al., 2014; Bedoya et al., 2015; Slimani et al., 2017). Furthermore, PT is widely employed by soccer coaches, because it requires little space or equipment, and uses short periods of training time (Ramirez-Campillo et al., 2014).

Plyometric training includes both forms of vertical or horizontal exercises and a combination of both (Ramirez-Campillo et al., 2018a). The SSC contributes more to vertical than to horizontal jump performance because the loading on the musculo-tendinous units is greater during the vertical jump, generating a larger stretching force, and allowing a greater use of elastic energy during the concentric phase (Maulder and Cronin, 2005; Kawamori et al., 2013). Recently, Ramirez-Campillo et al. (2015) have compared the effects of the three modalities of PT in young soccer players. The authors have showed that a combination of vertical and horizontal jumps produced greater improvements in strength than either vertical or horizontal stimuli alone. Consequently, it may be suggested that the optimal PT for young soccer players should include exercises in both axes, i.e., multi-directional PT.

As previously indicated, PT can improve the vertical jumping heights (Slimani et al., 2017; Ramirez-Campillo et al., 2018b), but also change of direction speed (CODS) (Ramirez-Campillo et al., 2015, 2018b), and postural control (Ramirez-Campillo et al., 2015) in young soccer players. However, the effects of multidimentional PT in under 21 year soccer players are not well known for dynamic postural control (DPC). The DPC has previously been associated with better soccer performance (Paillard et al., 2006). In fact, there is a relationship between sport expertise and postural skills (Paillard, 2019) and thus an improvement in postural abilities is likely to enhance sportive performance (Paillard, 2017a) especially if power strength is increased concomitantly (Paillard, 2017b). The improvements in DPC in both legs would not only enhance physical performance, but also would reduce the risk of lower-extremity injuries. Plisky et al. (2006) measure the DPC with the Star Excursion Balance Test (SEBT) in high school basketball players and these results indicated that boys players with an anterior right/left reach distance difference greater than 4 cm was significantly associated with lower extremity injury and that breeds 2.5 times more likely to sustain injury. Improvements in DPC may reflect a greater contraction force of the lower extremity muscles (Myer et al., 2006) and/or changes in proprioceptive and neuromuscular control (Hewett et al., 2002). However, although PT increases the contraction force of the lower extremity muscles, the effects of multi-directional PT on DPC are as yet unknown.

Consequently, the main objective of the present investigation was to study the effects of 6 weeks of in-season multi-directional PT on vertical jump height, CODS and DPC in under 21 soccer players. We hypothesized that multi-directional PT would improve these three indices of performance abilities.

## Materials and Methods

### Participants

**FIGURE 1:**
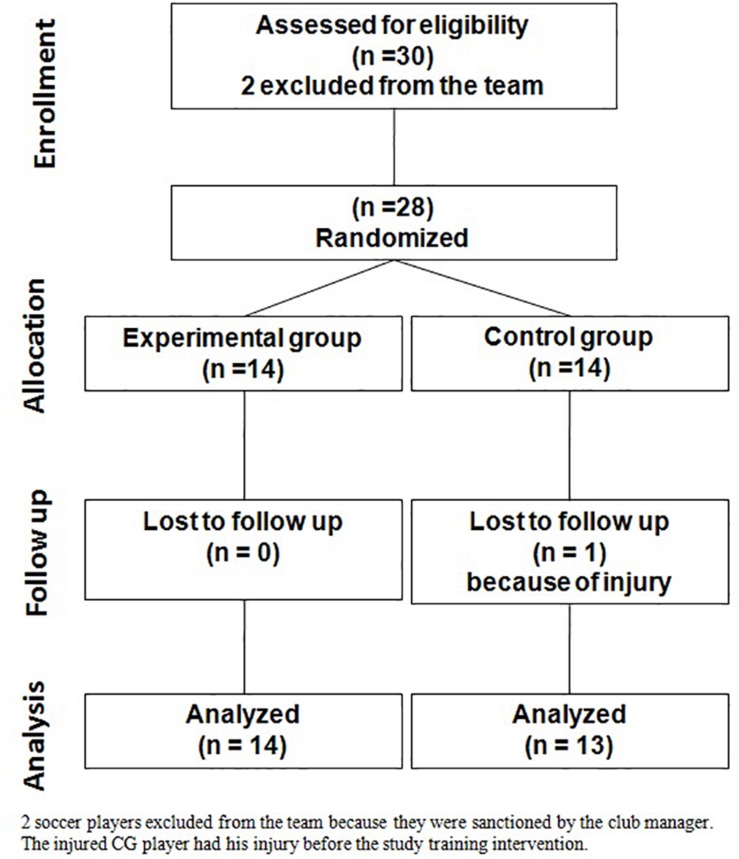
The consort diagram of the study.

Twenty-seven male soccer players participating in the Tunisian 3rd league championship took part in this study. The age for participants ranged from 18 to 20 years. They trained 5 days per week in the afternoon for ∼2 h per session. The representative sample size of our study was calculated with G^∗^Power 3.1 software. Preliminary analysis with an assumed Type I error of 0.05; Type II error of 0.20; and effect size = 0.25, was carried out. The results revealed that 24 participants were needed to reach 80% of statistical power. Therefore, we recruited few additional participants (*n* = 28) to take into account the potential withdrawing from the study during the training period. The participants (see consort diagram, Figure 1) were randomly allocated by computer to an experimental group (EG, *n* = 14) or a control group (CG, *n* = 14). After randomization of the groups and before the intervention, a soccer player in the CG has been injured because of a leg injury (CG, *n* = 13). There was no significant inter-group difference for age and anthropometric data (i.e., body height, leg length, body mass, and body mass index) (Table 1).

**TABLE 1:** Age and anthropometric data of participants.

	Control group (*N* = 13)	Experimental group (*N* = 14)	*p*-value	Cohen’s *d*
Age (yr)	19.0 ± 0.7	19.0 ± 0.9	0.867	0.16
Body height (m)	1.76 ± 0.06	1.76 ± 0.05	0.856	0.06
Leg length (m)	1.03 ± 0.04	1.03 ± 0.03	0.883	0.06
Body mass (kg)	69.2 ± 5.8	67.6 ± 5.9	0.508	0.27
Body mass index (kg/m^2^)	22.3 ± 1.7	21.9 ± 1.6	0.520	0.26
Goalkeeper (N)	1	2		
Defender (N)	3	4		
Midielder (N)	7	4		
Attacker (N)	3	4		

*N: number of soccer players.*

As the leg used to kick a ball has 100% agreement with the self-reported dominant leg (van Melick et al., 2017), the dominant leg was determined from following question: “If you would shoot a ball on a target, which leg would you use to shoot the ball?.” All participants were right leg dominant. None of the participants reported any recent history of hip, knee or ankle injury, or other pathological conditions of the lower limbs or lower back.

### Experimental Design

The study was approved by the Manouba University Ethics Committee. After being informed about the nature, risks, and benefits of the study, volunteers signed their informed consent in accordance with the Declaration of Helsinki. They were insured that they could withdraw from the trial without penalty at any time.

The study was performed over a 6 week period during March and April as part of an official competitive season that started in September and finished in June. Data were collected before and after the participants have completed the 6 week intervention period. Testing and multidirectional plyometric training (MPT) sessions (for EG) was integrated into the weekly training schedule. The MPT duration was of 23–33 min (the MPT program details are described in Table 2). The recovery between set repetitions was of approximately ∼15 s (Read and Cisar, 2001), with ∼60 s between series. All sessions of MPT were performed on the same artificial grass surface. The MPT replaced the first part of the regular soccer training volume (post-warm-up) into their regular soccer training sessions on every Tuesday and Thursday throughout the 6 week intervention. The CG training consisted of tactical drills (defensive drills, offensive drills, corner kicks situations, penalty kicks; ∼30 min), small-sided games with or without goal keeper and with or without change of soccer rules (e.g., one touch pass, only heading goals; ∼30 min), and simulated competitive games (∼40 min). While after the MPT, The EG continued the regular soccer training [small-sided games with or without goal keeper and with or without change of soccer rules (∼30 min) and simulated competitive games (∼40 min)] with CG.

**TABLE 2:** Multidirectional plyometric training program.

Week	Exercises	Directions	Number of jumps per exercise	Number of jumps per session	Total time
**1**	Alternating jumps (right-left leg) forward through the hoops	V-H	5 × 6	140	27
	Jumps with feet together and then separated in hoops	V-H-L	5 × 10		
	Jumps forward between barriers (45 cm)	V-H	5 × 4		
	Alternating jumps legs (right-left leg) on bench (50 cm)	V	5 × 8		
**2**	Jumps forward and back between ground markers with lateral displacement	V-H	5 × 16	160	23
	Squat jumping on a bench (30 cm)	V	5 × 8		
	Squat jumping on a bench (50 cm)	V	5 × 8		
**3**	Alternating lateral jumps (right-left leg)	V-H	6 × 16	168	25
	Lateral jumps over a bench (30 cm)	V-H	6 × 4		
	Alternating jumps legs (right-left leg) on bench (50 cm)	V	6 × 8		
**4**	Jumping, feet together between plots	V-H	6 × 10	180	25.5
	Combined jumps (front-lateral-back)	V-H-L	6 × 10		
	Lateral jumps between barriers (45 cm) without displacement	V-L	6 × 10		
**5**	Alternating lateral jumps (2 left, 2 right)	V-L	6 × 8	192	32
	Alternating jumps lateral (right-left leg) in the hoop	V-L	6 × 8		
	Alternating jumps, front-lateral between barriers (45 cm)	V-L	6 × 8		
	Squat jumps on a bench (50 cm)	V	6 × 8		
**6**	Alternating jumps (right-left leg) forward in the hoops	V-H	6 × 8	216	33
	Jumps with feet together and then separated in hoops	V-H-L	6 × 8		
	Jumps (4 front, 4 right side, 4 left side) between barriers (45 cm)	V-H-L	6 × 12		
	Squat jumping on a bench (50 cm) followed by drop jump on a bench (30 cm)	V	6 × 8		

*V, vertical; V-H, vertical-horizontal; V-L, vertical-lateral; V-H-L, vertical-horizontal-lateral.*

During the 3 days of testing soccer players performed regular training sessions with a reduction in the training content to have enough time for testing and not to increase the duration of the sessions. This training only consisted of tactical drills (∼30 min) and simulated competitive games (∼40 min). The tests were always performed after an appropriate warm-up, but before the soccer training sessions. Tests were performed by the same investigator in a fixed order over three different days. On the first test day, anthropometric measurements were completed, followed by vertical jumping height tests. The second test day was devoted to the evaluation of agility from CODS obtained during *T*-test. Finally, the balance was assessed from DPC during the third test day.

On the first test day, participants’ body height was measured using measuring rod anthropometric (version 216, Seca^®^ Hamburg, Germany). Leg length was measured by the same experienced investigator, from the most distal end of the anterior superior iliac spine to the most distal end of the lateral malleolus (Filipa et al., 2010), using a standard stainless steel tape measures with the participant lying supine on a plinth. Moreover, body mass was assessed with participants wearing light clothes and barefoot (Focus 9800 scale, EKS^®^, Gislaved, Sweden), then body mass index was calculated.

After these first measurements, a standardized warm-up was performed, then two different vertical jumping height tests (squat jump: SJ and countermovement jump: CMJ) were executed using an infrared photocell system (Optojump, Microgate^®^, Bolzano, Italy). The height of the jumps calculated from this system has previously been valid and reliable (Lehance et al., 2005). According to Ghoul et al. (2017), participants were asked to perform the SJ standing upright with good balance and the trunk as vertical as possible, feet parallel and shoulder-width apart, and hands on the hips throughout the test with a knee angle around 90°. The trial was not considered valid if any movement was perceived with the increased knee flexion at the start of the jump. For the CMJ, participants started from an upright standing position and made a preliminary downward movement by flexing the knees and hips, with a knee angle around 90° at the end of the countermovement (Ghoul et al., 2017). For both the SJ and the CMJ, the average of three trials (with 30-s of passive recovery between each, as proposed by Tabben et al. (2018) was used for analysis. The intra-class coefficients (ICCs) for three trials before and after intervention period with 95% confidence interval (95% CI) were 0.97 (95% CI = 0.95–0.99) for the SJ and 0.96 (95% CI = 0.94–0.98) for the CMJ, respectively.

On the second test day, the *T*-test was administered as described by Semenick (1990) and recently used by Chaabene et al. (2019) to assess the CODS. Participants sprinted forward 9.14 m to the first cone, touching its tip with their right hand, next shuffled 4.57 m left to the second cone, touching its tip with their left hand, then shuffled 9.14 m right to the third cone, touching its tip with their right hand, next shuffled 4.57 m left to the middle cone, touching its tip with their left hand before finally running backward to cross the starting/finishing line of 2 m wide. Times were recorded using an electronic timing gate (Photocells, Microgate^®^, Bolzano, Italy). The photoelectric cells were placed at a height of 0.7 m. Trials were deemed unsuccessful if participants failed to touch a designated cone, crossed their legs while shuffling or failed to face forward at all times. The average of three trials (separated by around 1-min passive recovery intervals) was used for analysis. The ICCs for three repeated trials before and after intervention period was 0.93 (95% CI = 0.90–0.96).

During the third test day, because it presents excellent reliability (Gribble et al., 2013), the SEBT was used as proxy to assess DPC. This functional unilateral balance test integrates a single-leg stance with maximum reach of the opposite leg (Hertel et al., 2000). The SEBT was performed with participants standing in the middle of a grid placed on the floor with eight lines extending at 45° increments from the center of the grid, as proposed by Olmsted et al. (2002). Moreover, according to these authors (Olmsted et al., 2002), eight lines on the grid were named in relation to the direction of reach relative to the stance leg: anterior (A), antero-lateral (AL), antero-medial (AM), medial (M), postero-medial (PM), posterior (P), postero-lateral (PL), and lateral (L). The protocol of Hertel et al. (2000) was followed, and the reach distances were normalized by dividing each excursion distance (in cm) by the participant’s leg length (in cm) and then multiplying the value obtained by 100. The average of the three trials was used for analysis. The ICCs for three trials before and after intervention period of the eight directions using dominant and non-dominant legs ranged from 0.90 to 0.94 (95% CI = 0.88–0.97).

Finally, to avoid possible variations in physical performances (Chtourou et al., 2012), all tests were performed at the same time of the day (4 to 6 p.m.) and under the same experimental conditions (22–26°C) at least 3 days after the most recent competition and/or the last MPT session. Moreover, familiarization sessions were held 2 weeks before the intervention period. Finally, a recommended by Haddad et al. (2014), the same standardized warm-up, which included 15 min of progressive running (two sets of 7 min 30 s) then dynamic stretching exercises (two repetitions of 30 s with a 15-s passive recovery for five muscle groups: knee extensors, knee flexors, ankle foot plantar flexors, hip adductors, and hip flexors) was performed before each test.

### Statistical Analysis

Data are expressed as means ± standard deviation. The normal Gaussian distribution was verified by the Shapiro–Wilk test. A two-way (two groups: CG vs. EG × 2 times: before vs. after) ANOVA for repeated measures was used to compare the data. The sphericity was checked by the Mauchley test and, when it was not met, the significance of *F*-ratios was adjusted according to the Greenhouse-Geisser or Huynh-Feldt procedures (according to the epsilon correction factor). To evaluate within-group pre-to-post performance changes, the Bonferroni *post hoc* was applied. The effect sizes (ES) were calculated to estimate the magnitude of the difference. The ES were determined by converting partial eta-squared values to Cohen’s *d* with the Excel spreadsheets. According to Cohen (Cohen, 1988) ES can be classified as small (0.00 ≤ *d* ≤ 0.49), medium (0.50 ≤ *d* ≤ 0.79), or large (*d* ≥ 0.80). Test-retest reliability was assessed using ICCs (Vincent, 1995). The level of significance was set at *p* ≤ 0.05. Statistical analysis was performed using STATISTICA version 10 (StatSoft^®^ France).

## Results

The anthropometric and descriptive data showed no significant inter-group difference at baseline (Table 1). Before the intervention, a soccer player in the CG has been injured because of a leg injury (CG, *n* = 13). During the intervention, the attendance rate in the regular training sessions for both groups was (CG: 93.8% and EG: 91.2%). The attendance rate in the PT sessions for EG was 93.5%. For the Pedro scale assessment, out of the 11 criteria, 2 were not applicable to our study setting (points 5 and 6). Indeed, it is not possible to have the participants and the coaches blinded to the intervention. For the 9 remaining points, the present study protocol scored 8 on the remaining 9 points of the Pedro Scale.

## SJ, CMJ and *T*-Test Performance

Descriptive values of Pre- and Post-tests for SJ, CMJ and *T*-Test are presented in Table 3. ANOVA demonstrated a significant group × time interaction for SJ (*F* = 6.03, *p* = 0.021), CMJ (*F* = 9.10, *p* = 0.006), and *T*-Test (*F* = 10.46, *p* = 0.002). The Bonferroni *Post Hoc* test demonstrated significant increase for the three tests in both group (EG and CG). For SJ (EG: *p* < 0.001; CG: *p* < 0.001), CMJ (EG: *p* < 0.001; CG: *p* = 0.005), and *T*-Test (EG: *p* < 0.001; CG: *p* = 0.02). Also, the percentage of change was greater for EG compared to CG [SJ (EG: Δ = 6.23 ± 2.58%); (CG: Δ = 4.51 ± 1.70%); CMJ (EG: Δ = 5.71 ± 3.32%); (CG: Δ = 2.82 ± 1.43%), and *T*-test (EG: Δ = −2.99 ± 1.47%); (CG: Δ = −1.15 ± 0.95%)].

**TABLE 3:** Vertical jump and *T*-Test performance before and after the intervention program.

Group test	Control (*N* = 13)			Bonferroni *post hoc* test	Experimental (*N* = 14)			Bonferroni *post hoc* test	ANOVA group × time		
	Pre	Post	% Δ	*p* value	Pre	Post	% Δ	*p* value	F	*p* value	Cohen’s *d*
SJ (cm)	27.57 ± 3.96	28.80 ± 3.99	4.51 ± 1.70	< 0.001*	27.80 ± 4.48	29.51 ± 4.30	6.23 ± 2.58	< 0.001*	6.03	0.021*	0.98
CMJ (cm)	29.82 ± 3.45	30.64 ± 3.34	2.82 ± 1.43	0.004*	30.45 ± 4.49	32.19 ± 4.69	5.71 ± 3.32	< 0.001*	9.10	0.006*	1.20
*T*-Test (s)	10.59 ± 0.55	10.47 ± 0.52	−1.15 ± 0.95	0.002*	10.59 ± 0.56	10.26 ± 0.50	−2.99 ± 1.47	< 0.001*	10.46	0.002*	1.43

* ≤ 0.05.

### DPC on the Dominant Leg Performance

Descriptive values of Pre- and Post-tests of DPC on the dominant leg performance are presented in Table 4. ANOVA demonstrated a significant group × time interaction for four axes [anterior (*F* = 5.48, *p* = 0.028), antero-lateral (*F* = 4.82, *p* = 0.038), postero-lateral (*F* = 4.82, *p* = 0.038), and medial (*F* = 6.77, *p* = 0.015)]. The Bonferroni *Post Hoc* test demonstrated significant increase in EG, but no significant change in CG in four axes [anterior (EG: *p* < 0.001, Δ = 2.27 ± 2.05%; CG: *p* = 0.62, Δ = 0.81 ± 1.41%), antero-lateral (EG: *p* < 0.001, Δ = 3.20 ± 3.12%; CG: *p* = 1.00, Δ = 0.86 ± 1.81%), postero-lateral (EG: *p* < 0.001, Δ = 1.96 ± 2.12%; CG: *p* = 0.82, Δ = 1.83 ± 2.52%), medial (EG: *p* < 0.001, Δ = 2.00 ± 2.31%; CG:*p* = 1.00, Δ = 1.67 ± 2.03%)]. However, for the rest of axes, there was no significant group × time interaction demonstrated.

**TABLE 4:** Dynamic postural control (SEBT) of the dominant leg performance before and after the intervention program.

Group axe	Control (*N* = 13)			Bonferroni *post hoc* test	Experimental (*N* = 14)			Bonferroni *post hoc* test	ANOVA group × time		
	Pre	Post	% Δ	*p* value	Pre	Post	% Δ	*p* value	F	*p* value	Cohen’s *d*
Anterior	71.2 ± 3.9	71.8 ± 3.8	0.81 ± 1.41	0.62	74.0 ± 3.8	75.7 ± 3.4	2.27 ± 2.05	<0.001*	5.48	0.028*	0.93
Antero-lateral	73.9 ± 4.5	74.5 ± 4.1	0.86 ± 1.81	1.00	73.9 ± 6.3	76.0 ± 5.2	3.20 ± 3.12	<0.001*	4.82	0.038*	0.87
Lateral	78.8 ± 4.0	79.4 ± 3.9	0.63 ± 0.82	_	80.9 ± 6.8	82.5 ± 6.6	1.92 ± 2.66	_	1.69	0.205	0.51
Postero-lateral	86.7 ± 3.3	87.3 ± 3.5	1.83 ± 2.52	0.81*	86.1 ± 4.0	87.8 ± 3.7	1.96 ± 2.12	<0.001*	4.82	0.038*	0.87
Posterior	84.7 ± 4.9	86.2 ± 3.9	0.99 ± 1.62	_	84.7 ± 6.4	86.9 ± 5.0	2.90 ± 2.48	_	0.85	0.365	0.36
Postero-medial	75.5 ± 7.0	76.2 ± 7.0	0.31 ± 1.09	_	74.1 ± 5.5	75.1 ± 5.1	1.41 ± 1.41	_	0.36	0.549	0.24
Medial	75.9 ± 3.2	76. 2 ± 3. 9	1.67 ± 2.03	1.00	75.7 ± 6.5	77.4 ± 5.1	2.00 ± 2.31	<0.001*	6.76	0.015*	1.04
Antero-medial	74. 3 ± 3. 6	75. 5 ± 4. 1	0.86 ± 1.81	_	74.0 ± 5.3	75.4 ± 5.1	1.98 ± 1.61	_	0.06	0.800	0.10

*: ≤0.05; _: No Bonferroni post hoc test.

### DPC on the Non-dominant Leg Performance

Descriptive values of Pre- and Post-tests tests of DPC on the non-dominant leg performance are presented in Table 5. ANOVA demonstrated a significant group × time interaction for three axes [lateral (*F* = 8.09, *p* = 0.009), postero-lateral (*F* = 11.92, *p* = 0.002), and medial (*F* = 5.84, *p* = 0.023)]. The Bonferroni *Post Hoc* test demonstrated significant increase in EG, but no significant change in CG in three axes: lateral (EG: *p* < 0.001, Δ = 2.17 ± 1.90%; CG: *p* = 1.00, Δ = 0.39 ± 1.18%), postero-lateral (EG *p* < 0.001, Δ = 1.97 ± 1.24%; CG: *p* = 0.07, Δ = 0.70 ± 0.74%), medial (EG: *p* < 0.001, Δ = 3.23 ± 2.31%; CG: *p* = 0.44, Δ = 1.10 ± 1.95%. However, for the rest of axes, there was no significant group × time interaction demonstrated.

**TABLE 5:** Dynamic postural control (SEBT) of the non-dominant leg performance before and after intervention program.

Group axe	Control (*N* = 13)			Bonferroni *post hoc* test	Experimental (*N* = 14)			Bonferroni *post hoc* test	ANOVA group × time		
	Pre	Post	% Δ	*p* value	Pre	Post	% Δ	*p* value	F	*p* value	Cohen’s *d*
Anterior	72.6 ± 3.4	72.6 ± 3.8	0.02 ± 2.84	_	74.4 ± 5.1	75.8 ± 4.7	1.80 ± 1.72	_	4.17	0.052	0.81
Antero-lateral	75.5 ± 5.3	76.4 ± 4.8	1.33 ± 2.44	_	79.4 ± 5.4	80.3 ± 4.9	1.24 ± 1.04	_	0.00	0.956	0.00
Lateral	80.3 ± 3.9	80.6 ± 3.6	0.39 ± 1.18	1.00	79.7 ± 4.3	81.4 ± 4.2	2.17 ± 1.90	<0.001*	8.09	0.009*	1.13
Postero-lateral	87.9 ± 3.2	88.5 ± 3.4	0.70 ± 0.74	0.07	87.0 ± 4.7	88.8 ± 4.2	1.97 ± 1.24	<0.001*	11.92	0.002*	1.38
Posterior	88.7 ± 3.7	88.3 ± 3.3	1.09 ± 1.53	_	88.4 ± 4.4	89.7 ± 3.7	1.69 ± 1.71	_	0.57	0.456	0.30
Postero-medial	78.5 ± 4.3	78.5 ± 4.4	−0.04 ± 1.96	_	80.2 ± 5.4	81.0 ± 5.6	1.16 ± 2.68	_	1.25	0.273	1.92
Medial	74.3 ± 4.2	74.3 ± 5.1	1.10 ± 1.95	0.43	73.6 ± 7.9	75.8 ± 7.1	3.23 ± 2.31	<0.001*	5.84	0.023*	0.96
Antero-medial	74.3 ± 4.2	75.1 ± 4.0	1.22 ± 2.41	_	73.9 ± 4.9	75.4 ± 4.8	2.33 ± 1.81	_	1.16	0.290	0.43

*: ≤0.05; _: No Bonferroni post hoc test.xs

## Discussion

In accordance with our original hypothesis, countermovement jump performance, CODS and DPC were all improved, although the amount and duration of multidirectional PT was relatively limited (2 sessions/week during 6 weeks).

Several previous studies have demonstrated the beneficial effects of PT on strength and/or CODS after programs ranging from 6 to 16 weeks (Ramirez-Campillo et al., 2014, 2015, 2018b; Sohnlein et al., 2014; Hammami et al., 2016; Lloyd et al., 2016; Negra et al., 2016). However, all of the programs previously performed were implemented in youth categories, whereas the present study focused on the responses of adult players (U-21). The present results confirm the effectiveness of a short dose of PT (6 weeks) in U-21 soccer players during the season. Moreover, the singularity of the present study is that it is unique by the combination of actions in the multi-directional planes to better meet the multi-directional needs of soccer activity.

In the present study, multi-dimensional PT has induced significant increases in vertical jumping heights in CMJ (Table 3). PT improves exercise performance that involves SSC of muscle–tendon units (Markovic and Mikulic, 2010). Hirayama et al. (2017) demonstrated that PT improved the SSC exercise performance by the optimization of muscle-tendon behavior of the agonists, associated with an alteration in the neuromuscular activity during SSC and an increase in tendon stiffness. Furthermore, a decrease in the neuromuscular activity of the antagonists during the braking phase appears to play an important role in this improvement. These findings can potentially explain why the present study showed a significant improvement of vertical jump with plyometric regime (CMJ, Δ = 5.71 ± 3.32%)].

Multi-directional plyometric training also improved performance of the CODS (Table 3), corroborating Miller et al. (2006) findings, which demonstrated that a 6 week in-season PT program improved CODS as measured by the *T*-Test. According to these authors (Miller et al., 2006), the improvement of CODS would be linked a reduced ground contact time, suggesting increased of strength and efficiency of movement.

The present study also evidenced improved DPC in four axes for the dominant leg (i.e., anterior, antero-lateral, postero-lateral, and medial; Table 4) and three axes for non-dominant legs (i.e., lateral, postero-lateral, and medial; Table 5). These gains could be related with the PT which develop the ability of soccer players improving their neuromuscular control by promoting anticipatory postural adjustments (Gantchev and Dimitrova, 1996; Asadi et al., 2015). Indeed, balance and stability challenges during PT can result in proactive and/or feed-forward adjustments that would adjust appropriate muscles contractions before the pitch-contact/landing (Marigold and Patla, 2002; Paillard, 2014). Furthermore, PT seems to result in improved sensitivity of afferent feedback pathway during exercise (Borghuis et al., 2008). Bedoya et al. (2015) recently suggested that the observed gains in performance could reflect various neuromuscular adaptations, such as an increased neural drive, improved inter-muscular coordination, changes in muscle size and architecture, and/or changes in single-fiber mechanics, as well as changes in muscle-tendon mechanical-stiffness (Markovic and Mikulic, 2010). Therefore, all these improvements could increase the performance and also potentially minimize the risk of injuries in soccer players (Chimera et al., 2004).

Although the present study points at the effectiveness of MPT, there remains a need to undertake a direct comparison between uni-, bi- and multi-directional plyometrics programs. Further, the study of larger samples of players over longer periods should allow an analysis of the extent of benefits by playing position. Finally, the improvements observed in the study could be due to the quality of the intervention (PT), and/or to a change in training load. Even though the training duration was the same in both groups, we did not accurately monitor training intensity during the experiment. Therefore, it is not possible to accurately determine from where the observed results were coming (intervention and/or training load effect).

In conclusion, the 6-week in-season multi-dimensional PT was effective to improve soccer-related specific physical qualities (i.e., countermovement jump performance, CODS and DPC). Furthermore, multi-dimensional PT has the potential to be appealing to coaches, as it requires little time while yielding valuable results in the physical preparation of U-21 soccer players.

## Data Availability Statement

The raw data supporting the conclusions of this article will be made available by the authors, without undue reservation, to any qualified researcher.

## Author Contributions

MJ and KC wrote the manuscript. JC contributed to the writing of the manuscript. TP performed the statistical analysis. NM, KC, and GB revised the manuscript.

## Conflict of Interest

The authors declare that the research was conducted in the absence of any commercial or financial relationships that could be construed as a potential conflict of interest.

## References

[B1] Asadi A. Saez de Villarreal E. Arazi H. (2015). The effects of plyometric type neuromuscular training on postural control performance of male team basketball players. *J. Strength Cond. Res.* 29 1870–1875. 10.1519/jsc.0000000000000832 25563677

[B2] Barros R. M. L. Misuta M. S. Menezes R. P. Figueroa P. J. Moura F. A. Cunha S. A. (2007). Analysis of the distances covered by first division brazilian soccer players obtained with an automatic tracking method. *J. Sports Sci. Med.* 6, 233–242. 24149334 PMC3786245

[B3] Bedoya A. A. Miltenberger M. R. Lopez R. M. (2015). Plyometric training effects on athletic performance in youth soccer athletes: a systematic review. *J. Strength Cond. Res.* 29 2351–2360. 10.1519/jsc.0000000000000877 25756326

[B4] Borghuis J. Hof A. L. Lemmink K. A. (2008). The importance of sensory-motor control in providing core stability: implications for measurement and training. *Sports Med.* 38 893–916. 10.2165/00007256-200838110-00002 18937521

[B5] Chaabene H. Negra Y. Moran J. Prieske O. Sammoud S. Ramirez-Campillo R. (2019). Plyometric training improves not only measures of linear speed, power, and change-of-direction speed but also repeated sprint ability in female young handball players. *J. Strength Cond. Res.* [Epub ahead of print]. 10.1519/jsc.0000000000003128 30946268

[B6] Chimera N. J. Swanik K. A. Swanik C. B. Straub S. J. (2004). Effects of plyometric training on muscle-activation strategies and performance in female athletes. *J. Athl. Train.* 39 24–31. 15085208 PMC385258

[B7] Chtourou H. Hammouda O. Souissi H. Chamari K. Chaouachi A. Souissi N. (2012). Diurnal variations in physical performances related to football in young soccer players. *Asian J. Sports Med.* 3 139–144. 23012632 10.5812/asjsm.34604 PMC3445640

[B8] Cohen J. (1988). *Statistical Power Analysis for the Behavioural Sciences.* Hillsdale: Erlbaum.

[B9] Di Salvo V. Baron R. Tschan H. Calderon Montero F. J. Bachl N. Pigozzi F. (2007). Performance characteristics according to playing position in elite soccer. *Int. J. Sports Med.* 28, 222–227. 10.1055/s-2006-924294 17024626

[B10] Filipa A. Byrnes R. Paterno M. V. Myer G. D. Hewett T. E. (2010). Neuromuscular training improves performance on the star excursion balance test in young female athletes. *J. Orthop. Sports Phys. Ther.* 40 551–558. 10.2519/jospt.2010.3325 20710094 PMC3439814

[B11] Gantchev G. N. Dimitrova D. M. (1996). Anticipatory postural adjustments associated with arm movements during balancing on unstable support surface. *Int. J. Psychophysiol.* 22 117–122. 10.1016/0167-8760(96)00016-5 8799774

[B12] Ghoul N. Tabben M. Miarka B. Tourny C. Chamari K. Coquart J. (2017). Mixed martial arts induces significant fatigue and muscle damage up to 24 hours post-combat. *J. Strength Cond. Res.* 33 1570–1579. 10.1519/jsc.0000000000002078 28658085

[B13] Gribble P. A. Kelly S. E. Refshauge K. M. Hiller C. E. (2013). Interrater reliability of the star excursion balance test. *J. Athl. Train.* 48 621–626. 10.4085/1062-6050-48.3.03 24067151 PMC3784363

[B14] Haddad M. Dridi A. Chtara M. Chaouachi A. Wong del P. Behm D. (2014). Static stretching can impair explosive performance for at least 24 hours. *J. Strength Cond. Res.* 28 140–146. 10.1519/JSC.0b013e3182964836 23615481

[B15] Hammami M. Negra Y. Aouadi R. Shephard R. J. Chelly M. S. (2016). Effects of an in-season plyometric training program on repeated change of direction and sprint performance in the junior soccer player. *J. Strength Cond. Res.* 30 3312–3320. 10.1519/jsc.0000000000001470 27135476

[B16] Hertel J. Miller J. S. Denegar C. R. (2000). Intratester and intertester reliability during the star excursion balance tests. *J. Sport Rehabil.* 9 104–116. 10.1123/jsr.9.2.104

[B17] Hewett T. E. Paterno M. V. Myer G. D. (2002). Strategies for enhancing proprioception and neuromuscular control of the knee. *Clin. Orthop. Relat. Res.* 402 76–94. 10.1097/00003086-200209000-00008 12218474

[B18] Hirayama K. Iwanuma S. Ikeda N. Yoshikawa A. Ema R. Kawakami Y. (2017). Plyometric training favors optimizing muscle-tendon behavior during depth jumping. *Front. Physiol.* 8, 16 10.3389/fphys.2017.00016 28179885 PMC5263151

[B19] Kawamori N. Nosaka K. Newton R. U. (2013). Relationships between ground reaction impulse and sprint acceleration performance in team sport athletes. *J. Strength Cond. Res.* 27 568–573. 10.1519/JSC.0b013e318257805a 22531618

[B20] Lehance C. Croisier J. L. Bury T. (2005). Optojump system efficiency in the assessment of lower limbs explosive strength. *Sci. Sport* 20 131–135.

[B21] Little T. Williams A. G. (2005). Specificity of acceleration, maximum speed, and agility in professional soccer players. *J. Strength Cond. Res.* 19 76–78. 10.1519/14253.1 15705049

[B22] Lloyd R. S. Radnor J. M. De Ste Croix M. B. Cronin J. B. Oliver J. L. (2016). Changes in sprint and jump performances after traditional, plyometric, and combined resistance training in male youth pre- and post-peak height velocity. *J. Strength Cond. Res.* 30 1239–1247. 10.1519/JSC.0000000000001216 26422612

[B23] Marigold D. S. Patla A. E. (2002). Strategies for dynamic stability during locomotion on a slippery surface: effects of prior experience and knowledge. *J. Neurophysiol.* 88 339–353. 10.1152/jn.00691.2001 12091559

[B24] Markovic G. Mikulic P. (2010). Neuro-musculoskeletal and performance adaptations to lower-extremity plyometric training. *Sports Med.* 40 859–895. 10.2165/11318370-000000000-00000 20836583

[B25] Maulder P. Cronin J. (2005). Horizontal and vertical jump assessment: reliability, symmetry, discriminative and predictive ability. *Phys. Ther. Sport* 6 74–82. 10.1016/j.ptsp.2005.01.001

[B26] Miller M. G. Herniman J. J. Ricard M. D. Cheatham C. C. Michael T. J. (2006). The effects of a 6-week plyometric training program on agility. *J. Sports Sci. Med.* 5 459–465. 24353464 PMC3842147

[B27] Myer G. D. Ford K. R. McLean S. G. Hewett T. E. (2006). The effects of plyometric versus dynamic stabilization and balance training on lower extremity biomechanics. *Am. J. Sports Med.* 34 445–455. 10.1177/0363546505281241 16282579

[B28] Negra Y. Chaabene H. Stoeggl T. Hammami M. Chelly M. S. (2016). Effectiveness and time course adaptation of resistance training vs. plyometric training in pre-pubertal soccer players. *J. Sport Health Sci.* [Epub ahead of print]. 10.1016/j.jshs.2016.07.008 PMC7749214 33308812

[B29] Olmsted L. C. Carcia C. R. Hertel J. Shultz S. J. (2002). Efficacy of the star excursion balance tests in detecting reach deficits in subjects with chronic ankle instability. *J. Athl. Train.* 37 501–506. 12937574 PMC164384

[B30] Paillard T. (2014). Sport-specific balance develops specific postural skills. *Sports Med.* 44 1019–1020. 10.1007/s40279-014-0174-x 24668292 PMC4072915

[B31] Paillard T. (2017a). Plasticity of the postural function to sport and/or motor experience. *Neurosci. Biobehav. Rev.* 72 129–152. 10.1016/j.neubiorev.2016.11.015 27894829

[B32] Paillard T. (2017b). Relationship between muscle function, muscle typology and postural performance according to different postural conditions in young and older adults. *Front. Physiol.* 8:585 10.3389/fphys.2017.00585 PMC5559497 28861000

[B33] Paillard T. (2019). Relationship between sport expertise and postural skills. *Front. Psychol.* 10:1428. 10.3389/fpsyg.2019.01428 31293483 PMC6603331

[B34] Paillard T. Noe F. Riviere T. Marion V. Montoya R. Dupui P. (2006). Postural performance and strategy in the unipedal stance of soccer players at different levels of competition. *J. Athl. Train.* 41 172–176. 16791302 PMC1472651

[B35] Plisky P. J. Rauh M. J. Kaminski T. W. Underwood F. B. (2006). Star Excursion Balance Test as a predictor of lower extremity injury in high school basketball players. *J. Orthop. Sports Phys. Ther.* 36 911–919. 10.2519/jospt.2006.2244 17193868

[B36] Rampinini E. Coutts A. J. Castagna C. Sassi R. Impellizzeri F. M. (2007). Variation in top level soccer match performance. *Int. J. Sports Med.* 28, 1018–1024. 10.1055/s-2007-965158 17497575

[B37] Ramirez-Campillo R. Alvarez C. Garcia-Hermoso A. Ramirez-Velez R. Gentil P. Asadi A. (2018a). Methodological characteristics and future directions for plyometric jump training research: a scoping review. *Sports Med.* 48 1059–1081. 10.1007/s40279-018-0870-z 29470823

[B38] Ramirez-Campillo R. Alvarez C. Gentil P. Moran J. Garcia-Pinillos F. Alonso-Martinez A. M. (2018b). Inter-individual variability in responses to 7 weeks of plyometric jump training in male youth soccer players. *Front. Physiol.* 9:1156. 10.3389/fphys.2018.01156 30177889 PMC6109752

[B39] Ramirez-Campillo R. Gallardo F. Henriquez-Olguin C. Meylan C. M. Martinez C. Alvarez C. (2015). Effect of vertical, horizontal, and combined plyometric training on explosive, balance, and endurance performance of young soccer players. *J. Strength Cond. Res.* 29 1784–1795. 10.1519/jsc.0000000000000827 25559903

[B40] Ramirez-Campillo R. Meylan C. Alvarez C. Henriquez-Olguin C. Martinez C. Canas-Jamett R. (2014). Effects of in-season low-volume high-intensity plyometric training on explosive actions and endurance of young soccer players. *J. Strength Cond. Res.* 28 1335–1342. 10.1519/jsc.0000000000000284 24751658

[B41] Read M. M. Cisar C. (2001). The influence of varied rest interval lengths on depth jump performance. *J. Strength Cond. Res.* 15, 279–283. 11710651

[B42] Reilly T. Williams A. M. Nevill A. Franks A. (2000). A multidisciplinary approach to talent identification in soccer. *J. Sports Sci.* 18 695–702. 10.1080/02640410050120078 11043895

[B43] Sarmento H. Marcelino R. Anguera M. T. CampaniÇo J. Matos N. LeitÃo J. C. (2014). Match analysis in football: a systematic review. *J. Sports Sci.* 32 1831–1843. 10.1080/02640414.2014.898852 24787442

[B44] Semenick D. (1990). Tests and measurements: the T-test. *Strength Cond. J.* 12 36–37.

[B45] Slimani M. Paravlic A. Bragazzi N. L. (2017). Data concerning the effect of plyometric training on jump performance in soccer players: a meta-analysis. *Data Brief* 15 324–334. 10.1016/j.dib.2017.09.054 29214194 PMC5712054

[B46] Sohnlein Q. Muller E. Stoggl T. L. (2014). The effect of 16-week plyometric training on explosive actions in early to mid-puberty elite soccer players. *J. Strength Cond. Res.* 28 2105–2114. 10.1519/jsc.0000000000000387 24476783

[B47] Tabben M. Ihsan M. Ghoul N. Coquart J. Chaouachi A. Chaabene H. (2018). Cold water immersion enhanced athletes’ wellness and 10-m short sprint performance 24-h after a simulated mixed martial arts combat. *Front. Physiol.* 9:1542. 10.3389/fphys.2018.01542 30443221 PMC6221982

[B48] van Melick N. Meddeler B. M. Hoogeboom T. J. Nijhuis-van der Sanden M. W. G. van Cingel R. E. H. (2017). How to determine leg dominance: the agreement between self-reported and observed performance in healthy adults. *PLoS One* 12:e0189876. 10.1371/journal.pone.0189876 29287067 PMC5747428

[B49] Vincent W. (1995). *Statistics in Kinesiology.* Champaign, IL: Human Kinetics.

